# Does Sex Matter? Temporal and Spatial Patterns of Cougar-Human Conflict in British Columbia

**DOI:** 10.1371/journal.pone.0074663

**Published:** 2013-09-11

**Authors:** Kristine J. Teichman, Bogdan Cristescu, Scott E. Nielsen

**Affiliations:** 1 Department of Biological Sciences, University of Alberta, Edmonton, Canada; 2 Department of Renewable Resources, University of Alberta, Edmonton, Canada; Federal University of Parana (UFPR)) – Campus Palotina, Brazil

## Abstract

Wildlife-human conflicts occur wherever large carnivores overlap human inhabited areas. Conflict mitigation can be facilitated by understanding long-term dynamics and examining sex-structured conflict patterns. Predicting areas with high probability of conflict helps focus management strategies in order to proactively decrease carnivore mortality. We investigated the importance of cougar (*Puma concolor*) habitat, human landscape characteristics and the combination of habitat and human features on the temporal and spatial patterns of cougar-human conflicts in British Columbia. Conflicts (*n* = 1,727; 1978–2007) involved similar numbers of male and female cougars with conflict rate decreasing over the past decade. Conflicts were concentrated within the southern part of the province with the most conflicts per unit area occurring on Vancouver Island. For both sexes, the most supported spatial models for the most recent (1998–2007) conflicts contained both human and habitat variables. Conflicts were more likely to occur close to roads, at intermediate elevations and far from the northern edge of the cougar distribution range in British Columbia. Male cougar conflicts were more likely to occur in areas of intermediate human density. Unlike cougar conflicts in other regions, cattle density was not a significant predictor of conflict location. With human populations expanding, conflicts are expected to increase. Conservation tools, such as the maps predicting conflict hotspots from this study, can help focus management efforts to decrease carnivore-human conflict.

## Introduction

Conflict between humans and wildlife is a widespread issue of concern in conservation of carnivores [1] [2]. Growing human populations and human encroachment into wildlife habitat, habitat loss and carnivore range expansion increase the likelihood of carnivore-human interactions. Understanding processes associated with temporal and spatial patterns of carnivore-human conflicts is essential to management planning [1].

Carnivore-human coexistence is dependent on maintaining low levels of conflicts [3], with high conflict levels potentially resulting in carnivore population sinks. An attractive sink is an area that animals may perceive as a favourable habitat but where risk of mortality is high [4]. Ideally, data on carnivore occurrence and human-caused mortality can be combined into habitat-based models that enable delineation of attractive sink-like habitats [4] [5]. However, in many cases conservation decisions need to be made in a timely fashion with relatively little information. A natural starting point is to simply describe and analyze spatial and temporal mortality patterns [6]. A more complex and rigurous approach is to develop predictive models for the spatial distribution of human-caused carnivore mortalities [7]. For example, models such as those developed in [8] are useful tools for identifying hotposts of livestock depredation by carnivores.

Loss of livestock to carnivores is a significant conservation challenge wherever livestock ranching practices overlap with carnivore range [9] [10]. In North America, all large carnivore species depredate on cattle (wolves [*Canis lupus*], [10]; grizzly bears [*Ursus arctos*], [11]; black bears [*Ursus americanus*], [12] and cougars [13]). Although radio telemetry-based monitoring of animals can reveal problem individuals such as cougars depredating on livestock [14], unmarked individuals, particularly sub-adult transients, may move into conflict hotspots. It may be these individuals that come into conflict with people [15] and livestock. Because depredation events are more likely to occur repeatedly in areas that have a history of conflict [9], predicting areas with high conflict potential would allow for more focused and preventative measures in conflict hotspots.

Several methods have been applied to reduce carnivore-human conflicts and these can be classified into lethal and non-lethal approaches. Historically, depredation events have been controlled with lethal management to reduce population densities, sometimes causing regional extirpations [16]. In addition, from the 1970’s to the 1990’s, translocation of ‘problem carnivores’ had been a standard management tool [17]. However, the loss or displacement of carnivores have poorly understood effects on population structure [18]. Removal of breeding adults may result in dependent young falling victim to starvation or killed by conspecifics moving into the vacant habitat [19]. Translocation may result in death by resident conspecifics, settlement and reproduction, or killing livestock in neighbouring areas [20]. Such conservation challenges can be minimized if a preventative approach is applied whereby management focuses on areas with high conflict probability [21].

Conflict mitigation is complicated by variability in behaviour between mammalian carnivore genders. For many solitary predators, males occupy larger home ranges [22] and have higher movement rates due to their evolutionary drive to search for females and defend their large territorial boundaries from intruding conspecifics [23] [24]. Also, males often disperse further distances compared to females [25] [26]. Seasonal differences in behaviour are also evident between males and females, for example, when females are raising young and have high energy demands thus spending more time searching and handling prey [27]. These differences may result in greater risk exposure of males in relation to humans such as when crossing roads or moving through ranchlands or wild-urban interfaces. Accounting for gender differences should therefore be considered when attempting to predict areas of high carnivore-human conflict.

Compared to other large carnivores in the Americas, cougars have lost the least of their historic range [28], with their persistence largely attributed to their secretive habits and solitary nature. Throughout their range, cougars come into conflict with people within areas dominated by livestock ranching [14] [29] [30] [31]. Furthermore, human encroachment in cougar habitat can result in cougar attacks on humans by dispersing sub-adult male cougars or emaciated, injured or diseased cougars that have difficulty capturing wild prey [15] [32]. The world’s greatest hotspot of cougar conflict is Vancouver Island located in British Columbia, Canada [15]. However, to date no study has quantified what specific habitat and human factors affect cougar conflicts for this area. It is suggested that British Columbia contains a stable cougar population estimated at 4,000–6,000 individuals, one of the highest of all states and provinces in North America [33]. Nonetheless, reliable estimation of cougar numbers is difficult and assigning ‘sustainable’ mortality rates that ensure cougar population persistence is not straighforward due to limited knowledge of cougar social systems and large-scale movements [18]. Thus, conservation and management decisions involving cougar populations will benefit from understanding cougar-human conflict patterns in order to minimize risk of conflict.

The objective of this study was to describe the temporal trend of cougar-human conflicts in British Columbia over the past thirty years and examine the spatial distribution of conflicts over a recent ten year period in relation to habitat and human features. We predicted that 1) most conflicts will occur in the summer which coincides with the greatest human presence on the landscape, 2) spatial models combining human and habitat features will be most supported and 3) male cougars will be more likely to get into conflict with people in association with human-related landscape features due to their wide ranging patterns and dispersal movements.

## Methods

### Study area

The study area encompasses cougar range in British Columbia, which coincides with the provincial extent excluding the extreme northern section of the province and most of the Pacific islands. Mean elevation is 1,184±499 m (mean ± sd) and 14.5% of the 765,703 km^2^ study area is protected under federal or provincial legislation. Mean human density is 4±68 individuals/km^2^, with the highest densities in the southern portion of the study area, which also has the highest road density. Cattle ranching is distributed mainly in the south-central and north-eastern parts of the province with mean cattle density at 0.3±10.8 cattle/km^2^.

### Temporal patterns of cougar-human conflict

Our data set consisted of thirty years of conflict records collected by the British Columbia Ministry of Environment from 1978–2007, undifferentiated by type of conflict. We therefore define cougar-human 'conflict' as any incident of cougar road mortality, depredation on livestock or attack on humans. Each record included geographically referenced location (UTM coordinates), date, and the type of conflict. We assessed temporal dynamics of conflict occurrence for each sex by season, differentiating between spring (March 21-June 19), summer (June 20-September 21), fall (September 22-December 20), and winter (December 21-March 20). Chi-square goodness of fit tests were used to assess differences in the total number of conflicts between seasons with expected seasonal conflict rates set at 25% of total recorded conflicts. We also tested for differences in the number of conflicts between sexes by performing chi-square goodness of fit tests for each season.

### Spatial patterns of cougar-human conflict

We used ten years of conflict data (1998–2007) and GIS spatial layers to estimate which variables adequately predict conflict and map the relative probability of cougar-human conflict. We did not use the entire thirty years of conflict data because accurate spatial information on conflicts was lacking for the 1978–1997 period and due to changes in landscape variables used to predict conflict hotspots. We carried out separate analyses for males and females without partitioning the data seasonally because of low sample sizes (female *n* = 222, male *n* = 222). To obtain a population level map of conflict potential, we computed the arithmetic mean between predicted values for the entire landscape, using the male and female predicted probabilites as inputs. Human and habitat characteristics at conflict locations were contrasted with those recorded at random locations to determine factors influencing conflict occurrence. Random locations were generated within British Columbia cougar range using Hawth's Tools [34] and a sampling intensity based on an estimated cougar home range size of 100 km^2^ [35] resulting in a total of 7,928 locations. Because we were interested in comparing conflict patterns between sexes, and conflicts involving cougars of unknown sex represented only 4.7% of total conflicts, these were excluded from the temporal and spatial analyses.

#### Predictors of cougar-human conflict

GIS variables selected to predict conflict locations were used in three sets of models, which included human variables, habitat variables and combined human-habitat variables respectively. Human variables hypothesized to influence conflict occurrence included human population density (http://sedac.columbia.edu/gpw), density and distance to roads (http://geogratis.cgdi.gc.ca/) and cattle density (http://fao.org). Densities and distances to nearest paved and unpaved roads were not included in models because they were correlated with the combined paved-unpaved respective road variables. Habitat variables hypothesized to affect cougar and prey density and thus conflict levels included elevation and terrain ruggedness (http://cits.rncan.gc.ca), land cover (http://bioval.jrc.ec.europa.eu/) reclassified to represent natural habitat occupied by cougars (conifer, deciduous, mixed forest and shrubland), forest edge density, distance to water combined as the minimum of the distances to nearest river and lake, protected area (http://geogratis.cgdi.gc.ca/) and distance to northern edge of the cougar range (http://iucnredlist.org/) (Table 1).

**Table 1:** Explanatory variables included in *a priori* human-cougar conflict models..

				Moving window (km^2^)	
Variable	Variable Abbreviation	Initial Units	Initial Data Range	Male	Female
*Habitat*					
Elevation	elev	m	–60−3,901	50	50
Elevation^2^	elev^2^	n/a			
Terrain Ruggedness Index	tri	n/a	0−2,709	50	50
Terrain Ruggedness Index^2^	tri^2^	n/a			
Land cover					
Conifer forest	conif	n/a	0 or 1	200	200
Deciduous forest	decid	n/a	0 or 1	200	200
Mixed forest	mixed	n/a	0 or 1	200	50
Shrubland	shrub	n/a	0 or 1	50	100
Distance to Water	diwat	km	0−26.8	50	50
Edge Density	edged	km/km^2^	0−0.9	50	100
Protected area	prot	n/a	0 or 1	200	200
Distance to northern cougar range edge	dinedge	km	0−1,020.2	200	200
*Human*					
Human Density	hdens	individuals/km^2^	0−4,862	200	50
Human Density^2^	hdens^2^	n/a			
Distance to Road	diroad	km	0−134.4	50	50
Density Roads	proadd	km/km^2^	0−2.1	50	50
Cattle Density	cattled	cattle/km^2^	0−2,023.4	200	200
Cattle Density^2^	cattled^2^	n/a			

Initial data range was changed to 0−100 for all variables as a result of moving window analyses.

Landscape characteristics in locations of cougar-human conflicts and at random locations were assessed using three moving windows in a GIS representing the average reported sizes of different cougar home ranges in British Columbia [35]. This approach followed Naves *et al*. [36] who applied it to their study of brown bears in Spain. The analysis resulted in three sets of landscape variables at the scales of 50 km^2^, 100 km^2^, and 200 km^2^ respectively. All layers were in raster format at 1 km^2^ grid cell size which also was employed in other studies [6] [8].

#### Modeling approach

We formulated *a priori* candidate models using biologically meaningful variables and used identical variable combinations for male and female models respectively. Models were fitted using logistic regression with a 1 (conflict) or 0 (random) outcome using STATA/SE-64. Competing sets of models included human models (*n* = 7), habitat models (*n* = 12), and combined human-habitat models. Human-habitat models were further split into simple (without cattle as a predictor, *n* = 36; with cattle, *n* = 48) and complex (without cattle but with interaction terms, *n* = 30; with cattle and interaction terms, *n* = 40) forms. All variables were screened for correlations (*r* > |0.7|) and only uncorrelated variables were used within the same model. We computed robust standard errors for all models to account for potential autocorrelation and/or heteroskedasticity in the regression error estimation. Fit of individual models was assessed by carrying out goodness of fit tests and calculating percentage deviance explained.

To rank models we computedAkaike’s Information Criterion corrected for small sample sizes (AICc) for each model and used the difference from the null model (▵AICc) and Akaike weights (*w*) in all model ranking procedures described below. First, using ▵AICc, three univariate models (one for each scale: 50 km^2^, 100 km^2^ or 200 km^2^) were run for each variable to select the most appropriate scale for the respective covariate. Once the suitable scale was identified separately for each sex, we ran the within-sets candidate models and ranked them for each set. Models that received support in the within-sets ranking procedure (▵AICc < 10) [37] were included in a final (between-sets) AICc ranking protocol that competed human, habitat and combined human-habitat models.

Simpler models are often most appropriate for prediction and easiest to understand, therefore most useful for management decisions. Because information criteria can be sensitive to sample size, to obtain optimal parsimony we re-ranked models included in the final AICc ranking by using a % deviance rule-of-thumb, whereby models needed to improve the percentage deviance explained by at least 1% for each additional parameter included in the model. By this rational, a model with 5 parameters explaining 30.5% of deviance ranked higher than one with 6 parameters explaining 31% of deviance, even though the first model may have had a higher AICc than the second one. This approach resulted in two top models for each sex, one as selected by ▵AICc and a second one as obtained based on deviance explained. We used variance inflation factors to check for collinearity between linear predictor variables in all four top models, eliminating highly collinear variables from the same model when necessary [38]. Finally, we investigated the predictive power of all top models by using 5-fold Cross Validation [39] wherein we partitioned the data into 5 bins and tested the model iteratively on 20% witheld data. Area-adjusted frequencies with significant Spearman-rank correlation coefficients (rho) were considered indicative of good predictive power.

## Results

**Figure 1:**
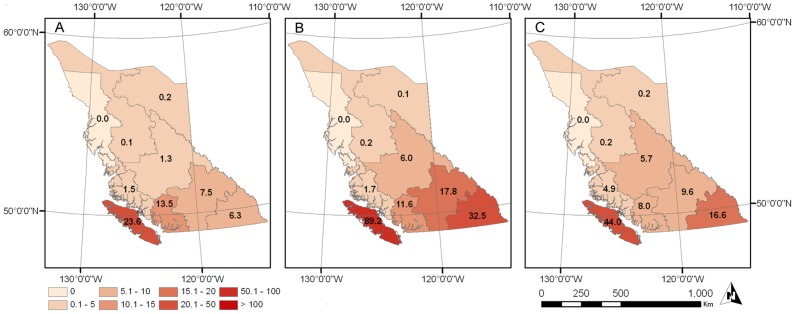
Temporal dynamics of cougar-human conflict in British Columbia, Canada across a period of three decades. (A) 1978–1987, (B) 1988–1997, (C) 1998–2007. Darkening shades of red indicate increase in number of conflicts. Numbers on maps represent mean conflict incidence per 10,000 km^2^ and are presented for each geographical region in the province.

A total of 1,727 conflicts involving cougars of known sex were recorded by the British Columbia Ministry of Environment over the thirty-year study period (female *n* = 847; male *n* = 880). The recent ten-year period (1998–2007) included a total of 444 conflicts with associated spatial information and known sex, resulting coincidently in a balanced data set for spatial analyses (female *n* = 222; male *n* = 222). Most conflicts occurred in central and southern British Columbia, with Vancouver Island having the largest number of conflicts of all regions within the province, a pattern which held during all three decades of data records (Figure 1).

### Temporal patterns of cougar-human conflict

**Figure 2:**
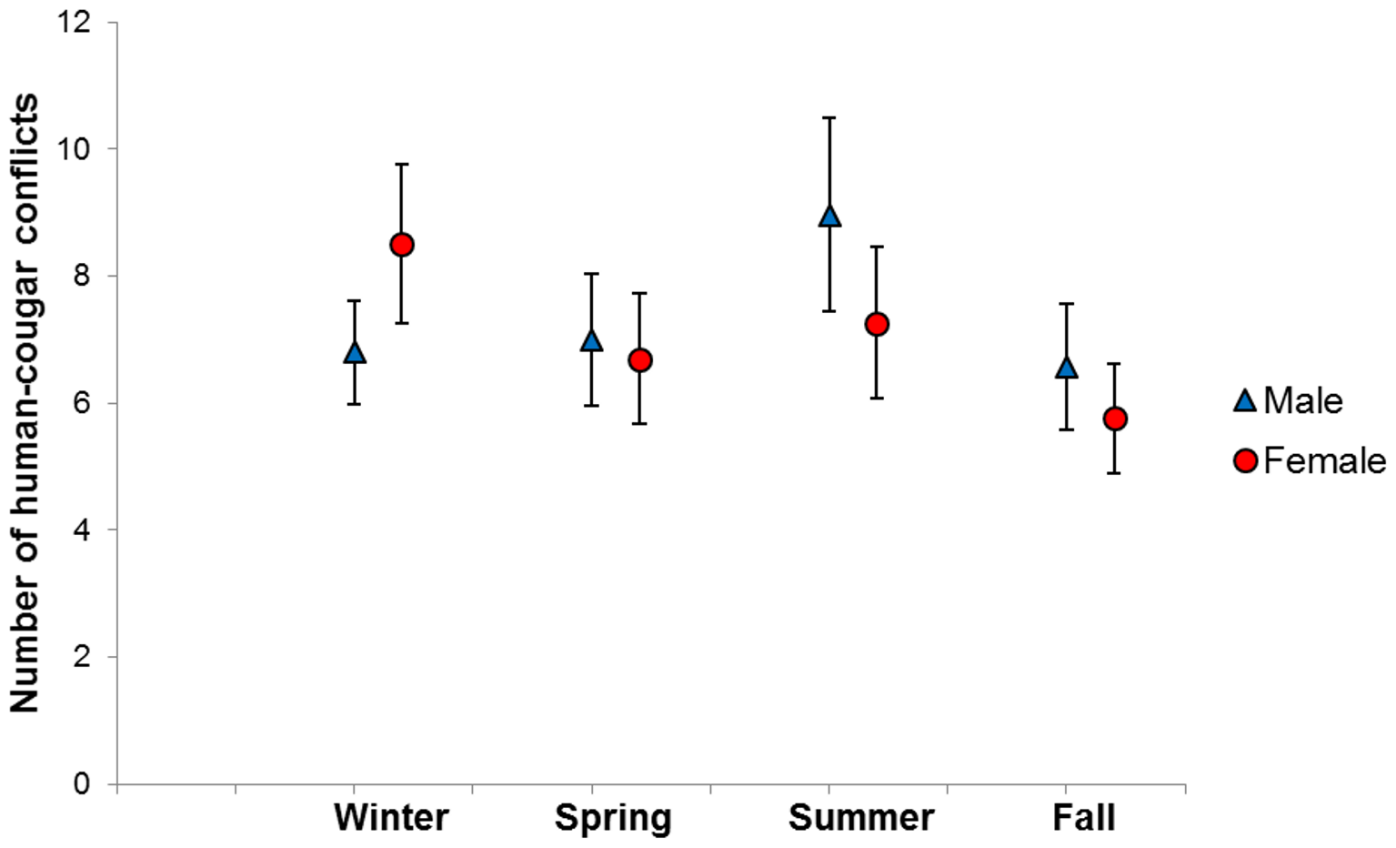
Seasonal variation in human-cougar conflict incidence in British Columbia based on thirty years of conflict data (1978–2007). Graphs represent means and error bars represent standard errors.

Minimum number of conflicts per unit area occurred during the first decade (1978–1987). Conflict numbers increased during the second decade (1988–1997) and finally decreased for the most recent decade (1998–2007), although this is still high relative to the first decade. Frequency of conflict differed between seasons for male (χ^2^ = 14.94, *df* = 3, *P* < 0.05) and female (χ^2^ = 16.66, *df* = 3, *P* < 0.05) cougars, with most conflicts recorded in summer and winter respectively (Figure 2). This pattern of conflict was also confirmed by contrasting frequencies of conflict between sexes on a seasonal basis. Conflict occurrence differed significantly between males and females for summer (χ^2^ = 5.34, *df* = 1, *P* < 0.05) and winter (χ^2^ = 5.67, *df* = 1, *P* < 0.05), but not for spring (χ^2^ = 0.20, *df* = 1, *P* = 0.66) and fall (χ^2^ = 1.56, *df* = 1, *P* = 0.21).

### Spatial patterns of cougar-human conflict

Models supported (▵AICc <10) in the final ranking procedure (between-sets) are reported in Table 2 (male) and Table 3 (female). All models had higher log pseudolikelihoods compared to the male and female null models respectively, including univariate models used to select the most appropriate scale for each variable. This finding confirmed that our *a priori* selected variables were adequate predictors of cougar-human conflict. For both sexes, models that incorporated human or habitat-only variables received no support (▵AICc ≥ 10) in the final (between-sets) ranking procedure. For both sexes, all human-habitat models competing in the final ranking received support, irrespective of whether interaction terms were present or not. The male and female top models selected via % deviance explained were from the combined human-habitat set, and so was the top ▵AICc - selected female model. The top ▵AICc - ranked male model, however, belonged to the set having human-habitat and interactions. As expected for both sexes, the top ▵AICc - selected model had a larger number of parameters than the top % deviance explained model. Models which included linear and squared terms for cattle density received less overall support than models with no cattle during initial ranking of the human model set, as well as during final ranking (between-sets).

**Table 2:** Summary of supported male cougar models for Human + Habitat, no cattle (a–e), Human + Habitat, with cattle (f–g), Human + Habitat Interaction, no cattle (h-n) and Human + Habitat Interaction, with cattle (o).

	Model Description	K	LL	AICc	ΔAICc	w	% dev. expl.
	null model (USE)	1	–1018.75	2039.6	802.6	0.00	0.00
a	hdens hdens^2^ diroad pprot dinedge elev elev^2^ conif diwat	10	–606.89	1241.1	4.1	0.05	40.43
**b**	* **hdens hdens^2^ diroad dinedge elev elev^2^**	**7**	–**613.35**	**1244.1**	**7.0**	**0.01**	**39.79**
c	hdens hdens^2^ diroad dinedge elev elev^2^ tri tri^2^	9	–609.75	1243.3	6.3	0.02	40.15
d	hdens hdens^2^ diroad dinedge elev elev^2^ pprot	8	–609.05	1238.6	1.6	0.16	40.22
e	hdens hdens^2^ diroad pprot dinedge elev elev^2^ mixed shrub diwat	11	–605.58	1242.3	5.2	0.03	40.56
f	cattled cattled^2^ hdens hdens diroad pprot dinedge elev elev^2^ conif diwat	12	–605.28	1245.7	8.7	0.00	40.59
g	cattled cattled^2^ hdens hdens^2^ diroad dinedge elev elev pprot	10	–607.56	1242.5	5.4	0.02	40.36
h	diroad dinedge elev elev^2^ mixed shrub diroad×mixed diroad×shrub	9	–608.81	1241.4	4.4	0.04	40.24
i	diroad dinedge elev elev^2^ mixed shrub diwat diroad×mixed diroad×shrub	10	–606.78	1240.9	3.8	0.05	40.44
j	diroad pprot dinedge elev elev mixed shrub diwat diroad×mixed diroad×shrub diroad×pprot	12	–602.11	1239.4	2.3	0.11	40.90
k	hdens hdens^2^ diroad dinedge elev elev^2^ pprot diroad×pprot hdens×pprot	10	–608.15	1243.6	6.6	0.01	40.30
**l**	** **hdens hdens diroad dinedge elev elev^2^ mixed shrub diroad×mixed diroad×shrub hdens×mixed hdens×shrub**	**13**	–**598.78**	**1237.0**	**0.0**	**0.35**	**41.22**
m	hdens hdens^2^ diroad dinedge elev elev^2^ mixed shrub diwat diroad×mixed diroad×shrub hdens×mixed hdens×shrub	14	–597.37	1238.9	1.9	0.14	41.36
n	hdens hdens^2^ diroad pprot dinedge elev elev^2^ mixed shrub diwat diroad×mixed diroad×shrub diroad×pprot hdens×mixed hdens×shrub hdens×pprot	17	–591.60	1243.8	6.8	0.01	41.93
o	cattled cattled^2^ diroad dinedge elev elev^2^ mixed shrub diwat cattled×mixed cattled×shrub diroad×mixed diroad×shrub	14	–601.38	1246.9	9.9	0.00	40.97

* Bold represents top model based on % deviance explained.

** Bold represents top model ranked using ΔAICc.

× refers to interaction between variables.

**Table 3:** Summary of supported female cougar models for Human + Habitat, no cattle (a–l), Human + Habitat, with cattle (m–r) and Human + Habitat Interaction, no cattle (s–y).

	Model Description	K	LL	AICc	ΔAICc	w	% dev. expl.
	null model (USE)	1	–1018.75	2039.6	660.3	0.00	0.00
a	diroad pprot dinedge elev elev^2^ conif diwat	8	–684.13	1388.8	9.5	0.00	32.85
**b**	* **diroad dinedge elev elev^2^**	**5**	–**688.12**	**1388.0**	**8.7**	**0.00**	**32.45**
c	diroad dinedge elev elev^2^ mixed shrub	7	–685.74	1388.9	9.6	0.00	32.69
d	diroad dinedge elev elev^2^ mixed shrub diwat	8	–682.32	1385.1	5.8	0.01	33.02
e	diroad pprot dinedge elev elev^2^ mixed shrub diwat	9	–682.27	1388.3	9.0	0.00	33.03
f	hdens hdens^2^ diroad pprot dinedge elev elev^2^ conif diwat	10	–677.77	1382.9	3.6	0.04	33.47
**g**	** **hdens hdens^2^ diroad dinedge elev elev^2^**	**7**	–**680.95**	**1379.3**	**0.0**	**0.25**	**33.16**
h	hdens hdens^2^ diroad dinedge elev elev^2^ pprot	8	–680.86	1382.2	2.9	0.06	33.17
i	hdens hdens^2^ diroad dinedge elev elev^2^ tri tri^2^	9	–679.64	1383.1	3.8	0.04	33.29
j	hdens hdens^2^ diroad dinedge elev elev^2^ mixed shrub	9	–678.95	1381.7	2.4	0.07	33.36
k	hdens hdens^2^ diroad dinedge elev elev^2^ mixed shrub diwat	10	–676.37	1380.1	0.8	0.17	33.61
l	hdens hdens^2^ diroad pprot dinedge elev elev^2^ mixed shrub diwat	11	–676.34	1383.8	4.5	0.03	33.61
m	cattled cattled200^2^ hdens hdens^2^ diroad pprot dinedge elev elev^2^ conif diwat	12	–676.22	1387.6	8.3	0.00	33.62
n	cattled cattled^2^ hdens hdens^2^ diroad dinedge elev elev^2^	9	–679.37	1382.6	3.3	0.05	33.31
o	cattled cattled^2^ hdens hdens^2^ diroad dinedge elev elev^2^ pprot	10	–679.32	1386.0	6.7	0.01	33.32
p	cattled cattled^2^ hdens hdens^2^ diroad dinedge elev elev^2^ tri tri^2^	11	–678.12	1387.3	8.0	0.00	33.44
q	cattled cattled^2^ hdens hdens^2^ diroad dinedge elev elev^2^ mixed shrub	11	–677.55	1386.2	6.9	0.01	33.49
r	cattled cattled^2^ hdens hdens^2^ diroad dinedge elev elev^2^ mixed shrub diwat	12	–674.96	1385.1	5.8	0.01	33.75
s	diroad pprot dinedge elev elev^2^ conif diwat diroad×conif diroad×pprot	10	–679.78	1386.9	7.6	0.01	33.27
t	diroad dinedge elev elev^2^ mixed shrub diroad×mixed diroad×shrub	9	–679.65	1383.1	3.8	0.04	33.29
u	diroad dinedge elev elev^2^ mixed shrub diwat diroad×mixed diroad×shrub	10	–676.44	1380.2	0.9	0.16	33.60
v	diroad pprot dinedge elev elev^2^ mixed shrub diwat diroad×mixed diroad×shrub diroad×pprot	12	–675.13	1385.4	6.1	0.01	33.73
w	hdens hdens^2^ diroad dinedge elev elev^2^ pprot diroad×pprot hdens×pprot	10	–680.34	1388.0	8.7	0.00	33.22
x	hdens hdens^2^ diroad dinedge elev elev^2^ mixed shrub diroad×mixed diroad×shrub hdens×mixed hdens×shrub	13	–673.63	1386.7	7.4	0.01	33.88
y	hdens hdens^2^ diroad dinedge elev elev^2^ mixed shrub diwat diroad×mixed diroad×shrub hdens×mixed hdens×shrub	14	–671.14	1386.4	7.1	0.01	34.12

Models for Human + Habitat Interaction, with cattle did not receive any support.

* Bold represents top model based on % deviance explained.

** Bold represents top model ranked using ΔAICc.

× refers to interaction between variables.

#### Males

**Figure 3:**
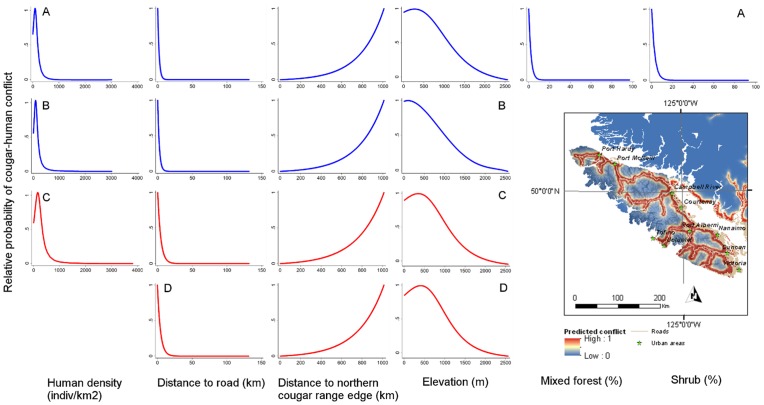
Predicted relative probability of cougar-human conflict in British Columbia. Predicted relative probability is based on variables from (A) Top male ▵AICc model, (B) Top % deviance explained male model, (C) Top ▵AICc female model, and (D) Top % deviance explained female model. Prediction for males are in blue and for females in red. Inset map illustrates conflict predictions for Vancouver Island, with elevation set as transparent in the background. For the inset only the top ▵AICc population-level model predictions are shown due to closely matching predictions with the correponding top % deviance explained model.

**Figure 4:**
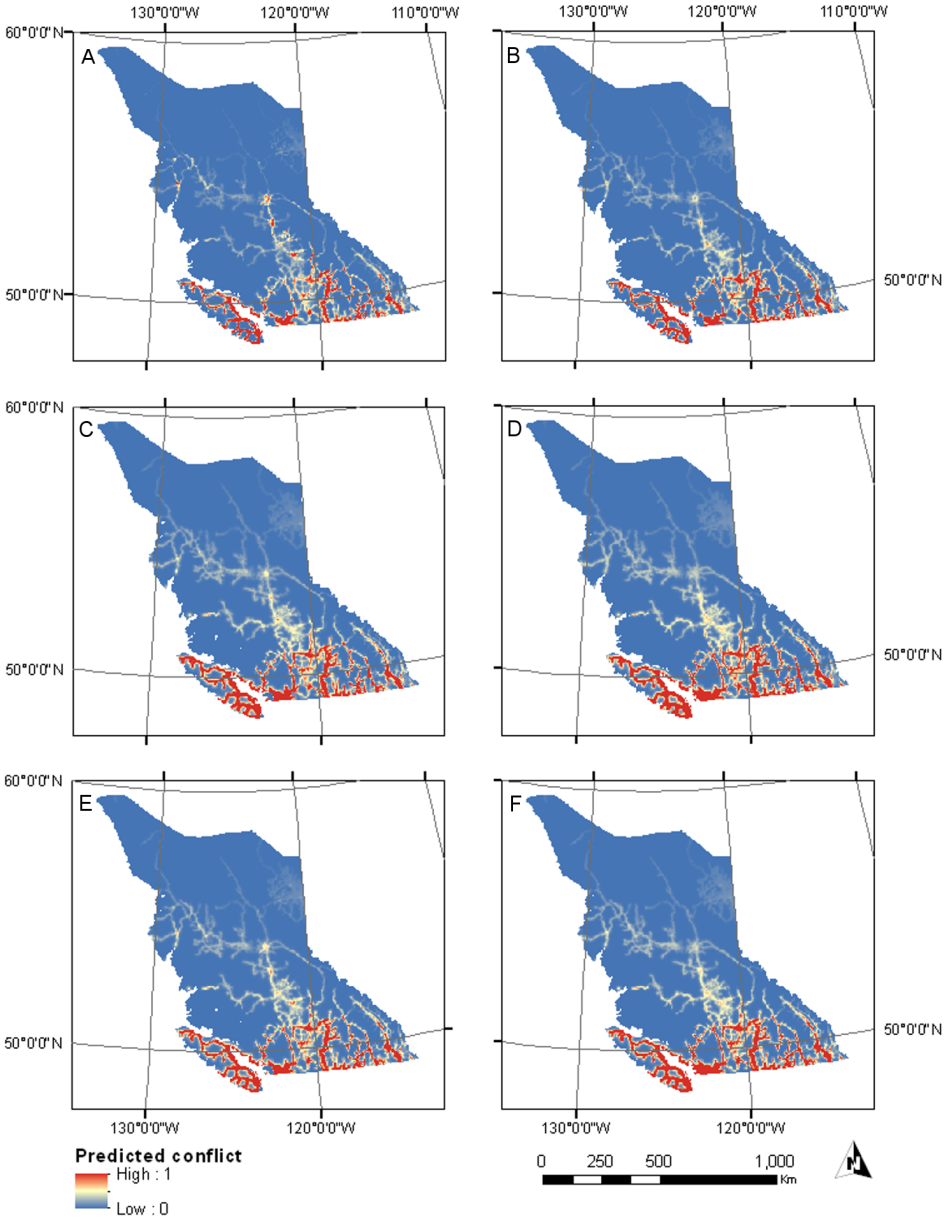
Top model predictions for the relative probability of cougar-human conflict in British Columbia. (A) Top ▵AICc male model, (B) Top % deviance explained male model, (C) Top ▵AICc female model, (D) Top % deviance explained female model, (E) Top ▵AICc population-level model, (F) Top % deviance explained population-level model. Predictions were based on conflict data for 1998–2007 (female *n* = 222; male *n* = 222).

Based on the top ▵AICc - selected model, male cougar conflicts were more likely to occur on/near roads, far from the northern cougar range in British Columbia, and at intermediate elevations in areas with high proportion of mixed forest (Figure 3a, Table 4). However, based on the interaction term between proportion mixed forest and distance to roads, mixed forest areas far from roads were less likely to have conflict. This model had 13 parameters, an AICc weight of 0.35 and explained 41.2% of the deviance. The model had good predictive power (mean ρ = 0.84, range 0.80−0.93) and its resulting predictions mapped in Figure 4a show conflict risk concentrated along roads and southern British Columbia, as well as high conflict for human populated areas in the central part of the province.

**Table 4:** Estimated cougar-human conflict location coefficients for male cougars in British Columbia.

Best ΔAICc Model					Best % Deviance Explained Model				
*Human + Habitat Interaction Model*					*Human + Habitat Model*				
Variable	Coef.	SE	CI		Variable	Coef.	SE	CI	
hdens^a^	10.101	7.702	–0.499	25.196	hdens^a^	13.339	5.490	2.578	24.100
hdens^2^ a	–0.059	0.032	–0.121	0.004	hdens^2^ a	–0.076	0.031	–0.136	–0.016
diroad^a^	–0.231	0.069	–0.366	–0.096	diroad^a^	–0.439	0.089	–0.613	–0.265
dinedge^b^	0.434	0.038	0.370	0.517	dinedge^b^	0.406	0.033	0.341	0.471
elev^a^	0.490	0.711	–0.903	1.884	elev^a^	0.077	0.641	–1.180	1.334
elev^2^ b	–0.093	0.040	–0.172	–0.015	elev^2^ b	–0.074	0.037	–0.147	0.002
mixed^a^	36.408	9.093	18.585	54.230					
shrub^a^	17.136	11.564	–5.529	39.802					
hdens×mixed^a^	0.024	0.205	–0.377	0.425					
hdens×shrub^a^	–0.093	0.329	–0.738	0.552					
diroad×mixed^b^	–2.210	0.599	–3.390	–1.040					
diroad×shrub^b^	–1.530	0.845	–3.190	0.125					

a Estimated coefficients, standard errors and confidence intervals reported at 1000 times their actual values.

b Estimated coefficients, standard errors and confidence intervals reported at 100,000 times their actual values.

Based on the top % deviance explained - selected model, conflicts were more likely to occur in areas of high human density, on/near roads, far from the northern cougar range edge and at intermediate elevations (Figure 3b, Table 4). The model included 6 parameters, and although it had an AICc weight of 0.01, it explained 39.8% of the deviance. This model also had good predictive power (mean ρ = 0.75, range 0.70−0.87) and its resulting predictions mapped in Figure 4b show conflict hotspots concentrated in southern British Columbia, along road corridors and less so in dense human areas compared to the previous model.

#### Females

According to the top ▵AICc - selected model, female cougar conflicts were more likely on/near roads, far from the northern cougar range in British Columbia and at intermediate elevations (Figure 3c, Table 5). This model had 6 parameters, an AICc weight of 0.25 and explained 33.2% of the. The model also included human density but the confidence intervals of the coefficient estimate for this variable overlapped zero. The predictive performance of this model was good (mean ρ = 0.85, range 0.66−0.96) and its predictions in Figure 4c are similar to that of the % deviance model. The one exception is that areas with high human density are not predicted to have high conflict, a difference due to the quadratic form of the human density variable. When compared to the male ▵AICc - selected model, the corresponding female model predicted larger conflict hotspots on/near roads, but fewer conflicts in human populated areas in central British Columbia.

**Table 5:** Estimated cougar-human conflict location coefficients for female cougars in British Columbia.

Best ΔAICc Model					Best % Deviance Explained Model				
*Human + Habitat Interaction model*					*Human + Habitat Model*				
Variable	Coef.	SE	CI		Variable	Coef.	SE	CI	
hdens^a^	6.513	3.782	–0.900	13.925	diroad^a^	–0.232	0.051	–0.333	–0.131
hdens^2^ b	–2.060	1.420	–4.840	0.724	dinedge^b^	0.395	0.031	0.335	0.455
diroad^a^	–0.228	0.052	–0.330	–0.126	elev^a^	1.067	0.606	–0.121	2.255
dinedge^b^	0.386	0.031	0.326	0.446	elev^2^ b	–0.139	0.035	–0.208	–0.070
elev^a^	0.808	0.619	–0.405	2.020					
elev^2^ b	–0.123	0.035	–0.191	–0.055					

a Estimated coefficients, standard errors and confidence intervals reported at 1000 times their actual value.

b Estimated coefficients, standard errors and confidence intervals reported at 100,000 times their actual value.

Based on the top % deviance explained-selected model, conflicts were more likely to occur on/near roads, far from the northern cougar range edge and at intermediary elevations (Figure 3d, Table 5). The model included 4 parameters, and although it had an AICc weight < 0.01, it explained 32.5% of the deviance. This model also had good predictive power (mean ρ = ρ0.85, range 0.66−0.93) and its predictions shown in Figure 4d illustrate conflict hotspots along road corridors, with more extensive conflict hotspots on/near roads than in the case of the top % deviance explained male model.

The ▵AICc - selected model had the highest mean Spearman correlation coefficient among the top two male models, which is indicative of slightly better predictive accuracy for the relative probability of occurence of a conflict location. In the case of the top two female models, the ▵AICc and % deviance explained selected models had similar predictive accuracies as illustrated by equal mean rho. Mean relative probabilities of conflict corresponding to population-level conflict risk are mapped in Figure 4e and Figure 4f.

## Discussion

Conflicts were lowest in 1978–1987 potentially reflecting low cougar population densities as a result of a North American-wide attempt at reducing top predators [40]. However, cougar density estimates are hard to obtain and relating them to incidence of conflict can be questionable [41]. For example, high conflict incidences have been related to low or declining cougar populations [42] [43]. More recently, human encroachment and habitat fragmentation may have caused the increased conflict incidence observed during 1988–1997 and 1998–2007, although to a lesser extent in the latter decade. Possible causes for the recent downward trend in conflict include increased public awareness as a result of campaigns for mitigating carnivore-human conflict [44] or lowered cougar movement rates within their home ranges in response to increased deer populations, which is the opposite of increased carnivore movements at low prey densities [45].

The seasonal differences in conflict occurrence between male and female cougars likely are due to underlying biological processes characteristic of the two sexes. Conflicts involving males occurred more in the summer, which is the period with the highest human recreational use, although our data did not allow explicit testing of human recreation effects on conflict occurrence. Although females in this species are induced ovulators [46] and reproduction can be seasonal due to naturally fluctuating photoperiods, most births in North America occur during summer months [47] [48] [49]. Females have high energetic requirements during gestation and lactation [50] [51] and kill rates are highest for females with kittens than for any other cougar sex/reproductive class [52]. Accordingly, conflicts for females peaked in winter when cougars are often accompanied by fully dependent kittens. To fulfill their dietary requirements during this time, females may be active for longer periods, have different movement patterns and hunting behaviour, such as using risky areas near roads where ungulate prey may be concentrated for foraging [53]. In addition, females may be scavenging to supplementary feed [54], which would make them more susceptible to conflict if they exploit ungulate road kills.

Spatially, cougar-human conflicts were most likely to occur in southern and central British Columbia, far from the northern edge of the species’ distribution. Although prey mortality risk may increase with harsh snow conditions [55], cougars may not be effective predators at high snow cover [56], which may explain their northerly range boundary in British Columbia. Their distributional range follows closely the distribution of their preferred prey, which in Canada are whitetail deer (*Odocoileus virginianus*) and mule deer (*Odocoileus hemionus*) [52] [57]. Conflicts occurred mostly at intermediate elevations, as high mountain areas have low vegetative productivity and thus little availability of cougar prey [58]. With climate warming deer populations are expected to expand northwards [59] and upwards, which may in the future lead to cougar range expansion and increased conflict at higher latitudes and altitudes. Cougars have already been shown to persist at high elevations in the Andes [60].

Our top models for cougar-human conflict included distance to northern range edge and elevation habitat variables which are indicative of broad scale habitat preferences. Finer scale habitat information such as forest edge density or distance to nearest water, both good deer habitat predictors [61] [62], were not present in the best models. From the perspective of predictive value, this is excellent as broader scale environmental data are readily available for many regions. However, one of the top male models included mixed forest and shrub, which are preferred cougar habitat because of deer presence and stalking cover. These two habitat variables do not compromise a model’s predictive value because vegetative land cover data are now widely available. Conifer forest did not predict conflict well likely because needleleaf forest cover is extensive across Brisith Columbia and likely supports lower deer densities, especially relative to browse-rich mixed forest and shrub.

Although cougar distribution in human-modified landscapes is determined by habitat requirements as well as avoidance of people [63], encounter risk may be increased by human expansion into cougar habitat. All top models showed that conflicts were more likely to occur on/near roads irrespective of cougar sex. Roads facilitate human access into cougar areas, and if placed in good cougar habitat may increase vehicular collisions because of high incidence of cougar road crossings. Roads that cross through areas with visibility obstructions may be particularly risky for cougars and other wildlife because they decrease the drivers’ reaction time to a crossing animal. Roads thus represented ubiquous conflict hotspots [64] whereas human density was a good conflict predictor for males but not necessarily for females. Human density was present in both the top male models and in the ▵AICc – selected female model but was absent from the % deviance explained – selected model for females. During dispersal, male cougars encounter human inhabited areas where they are likely to get into conflict. These findings conform with worldwide patterns of widlife-human conflict in relation to roads [65] [66] and male-biased conflict in human-use areas [51] [67].

Cougars depredate on livestock [13] [14] and we expected an increased probability of conflict in British Columbia in areas with high cattle density. In particular, we suspected cattle density would be an important predictor of cougar conflict for males because transients are more likely to depredate livestock [68]. For both sexes conflicts were not predicted to occur in areas of high cattle density, possibly because of easy access to expanding white-tailed deer populations [69].

Carnivore conflict mitigation involves efforts at different scales and we recognize that more accurate conflict predictions could be obtained at the local scale (e.g., the extent of a township) if finer scale variables were utilized. For example, our forest edge density variable used as a surrogate for deer distribution was based on forest versus other land cover types and did not include edge density based on local disturbances such as cutblocks, roads, and seismic lines. Our mapped predictions are thus most informative for analyzing conflict probability at regional or continental levels, typically used in large scale conservation planning strategies. Future Resource Selection Function (RSF) modeling applications of conflict data could benefit from incorporating more refined information, for example records of animal age, and female lactation data gathered during carcass inspection. Reproductive status influences movement behaviour and risk taking in all wildlife species [70]. Such information could improve studies that did not consider demography in mortality predictions.

## Conclusions

Carnivore-human conflict is a global conservation challenge that is expected to be on the rise with expanding human populations. In the case of cougars in British Columbia, distance to roads was the most important predictor of conflict locations out of all the potential human influences present in top models. This pattern is eloquently illustrated by the predicted conflict risk for Vancouver Island, the world’s largest cougar conflict hotpsot (Figure 3 inset). Fewer parameters were required to explain conflict patterns for females than for males, reflecting the higher variability of risk exposure by males during their wide ranging excursions. Decreasing current speed limits in predicted conflict hotpsots and introduction of cougar road crossing signs may help to reduce road mortality by creating more time for drivers to react to animals on roads, while simultaneously raising awareness of cougar presence. Because road mortality is common for many large carnivores [71] [72] [73] [74] [75] [76], when possible road access [74] and road density should be limited [77].

The predictive capacity for carnivore-human conflict may be increased by including reproductive status and age structure in conflict modeling. On the other hand, habitat data are increasingly available and we have shown that broad habitat patterns can be adequate predictors of cougar-human conflict. Predictions of potential areas that are likely to have cougar-human conflicts can help target management strategies that reduce cougar mortalities. With reduced occurrences of cougar-human conflicts, people are more likely to accept and thus coexist with cougars in a shared landscape such as southern British Columbia.
